# The Role of the Neutrophil-to-Lymphocyte Ratio and Platelet-to-Lymphocyte Ratio as Markers of Disease Activity in Ankylosing Spondylitis

**DOI:** 10.7759/cureus.6025

**Published:** 2019-10-29

**Authors:** Alam Zeb, Sadia Khurshid, Saira Bano, Uzma Rasheed, Shazia Zammurrad, Muhammad Sufyan Khan, Wajahat Aziz, Saira Tahir

**Affiliations:** 1 Rheumatology, Pakistan Institute of Medical Sciences, Islamabad, PAK

**Keywords:** nlr, plr, ankylosing spondylitis, basdai

## Abstract

Background

Ankylosing spondylitis (AS) is a chronic rheumatological condition affecting sacroiliac joint and spine and occurs more often in younger patients than in the elderly population.

Objective

The purpose of the study was to determine the association of the neutrophil-lymphocyte ratio (NLR) and platelet-lymphocyte ratio (PLR) with the disease activity of AS.

Methodology

This case-control study was conducted in the rheumatology department at the Pakistan Institute of Medical Sciences (PIMS) hospital in Islamabad from September 2018 to July 2019. The study consisted of two groups of 59 patients per group. We assessed a full blood count with erythrocyte sedimentation rate (ESR) for each participant using the PIMS hospital laboratory. NLR and PLR were calculated.

Results

The mean age of the participants in the control group and the cases group was the same (32 ± 4 years). The control group NLR was 1.30 ± 0.16, the PLR was 94.98 ± 17.96, and the ESR was 16.88 ± 3.76 mm/hour. For the cases group, the NLR was 3.08 ± 0.91, the PLR was 171.50 ± 38.06, and the ESR was 29.30 ± 9.20 mm/hour. There was a significant increase in cases for NLR, PLR, and ESR as compared to control samples (p<0.05). The mean Bath Ankylosing Spondylitis Disease Activity Index (BASDAI) score of participants with active diseases was 5.91±1.28. In the same group, the mean ESR was 27.65 ± 9.07 mm/hour, the NLR was 3.46 ± 0.80, and the PLR was 184.39 ± 36.13. For those in the inactive disease group, the mean BASDAI score was 2.84 ± 0.46, the ESR was 33.42 ± 8.48 mm/hour, the NLR was 2.17 ± 0.37, and the PLR was 139.71 ± 26.05. NLR and PLR were significantly higher in the active disease group (p<0.05).

Conclusion

NLR and PLR are good markers of inflammation in AS patients, and higher values indicate more active disease activity.

## Introduction

Ankylosing spondylitis (AS) is a chronic, systemic inflammatory disease that mainly involves the sacroiliac joint and spine. It represents the archetype of spondyloarthropathies. Its etiology is unknown but is associated with the presence of human leukocyte antigen B27 in 90% to 95% of cases [1]. The worldwide prevalence of AS is 0.5% to 1 %, while that in northern and southern Pakistan is 0.1 to 0.9 per 1000 people [2,3]. It mostly affects male patients [2,3].

AS can be diagnosed clinically and radiographically using modified New York diagnostic criteria. There is no specific diagnostic test. The Bath Ankylosing Spondylitis Disease Activity Index (BASDAI) is used to assess disease activity in AS. Erythrocyte sedimentation rate (ESR) and other acute-phase reactants are usually tested in AS patients. However, ESR and other acute-phase reactants are not related to disease activity, and ESR changes are seen in <50% of patients [4,5]. Interleukin-6 (IL6) and tumor necrosis factors (TNFs) are considered markers of inflammation in AS but are not routinely tested in AS patients [6].

Routine investigations for rheumatological diseases usually involve a full blood count (FBC) assessment, including total leukocyte count and platelet count. The white blood cell (WBC) and its differential counts undergo relative changes in systemic inflammation. Neutrophil and platelet levels increase with inflammation, and lymphocyte levels decrease in autoimmune diseases. The neutrophil-to-lymphocyte ratio (NLR) and platelet-to-lymphocyte ratio (PLR) are markers of different inflammatory conditions. A high NLR has high sensitivity and specificity for acute appendicitis, and high NLR and PLR are common to chronic autoimmune thyroiditis [7,8].

Neutrophil and platelet counts increase with inflammation, and lymphocyte counts decrease in autoimmune diseases. NLR and PLR are indicators of disease activity in systemic lupus erythematosus (SLE) patients [9]. High NLR is found in lupus nephritis [10]. Raised NLR and PLR indicate active rheumatoid arthritis (RA) [11]. NLR and PLR are indicators of inflammation and disease activity in SLE, RA, and malignancy [12,13].

The purpose of this study was to determine the relationship of NLR and PLR with disease activity of AS. NLR and PLR are calculated from FBC, which is cost-effective and time-saving. All other markers such as IL6, IL1b, TNF, and leptin levels are not feasible in a country like Pakistan for monitoring disease activity. NLR and PLR can be used for monitoring the efficacy of treatment in the future.

## Materials and methods

We conducted this case-control study in the rheumatology department at the Pakistan Institute of Medical Sciences (PIMS) hospital in Islamabad. The sample size was calculated by using the World Health Organization sample size calculator. The study was approved by the hospital ethical committee. From September 2018 to July 2019, 59 patients with AS who fulfilled the inclusion criteria were assigned to the study group, and 59 healthy subjects were assigned to the control group. Patients were included in the study if they were older than 18 years and had AS according to the American College of Radiology criteria. Patients were excluded if they had evidence of infections, a WBC count of >12000 or <3000, had thrombocytopenia or platelet count <140,000 or had a history of malignancy. After providing written informed consent, each patient was assessed clinically, and the BASDAI score was calculated. FBC with ESR was done at the PIMS hospital laboratory, and we calculated the NLR and PLRs.

Statistical analysis

We used IBM SPSS Statistics for Windows, Version 23.0 (IBM Corp, Armonk, NY) for storing and analyzing data. Mean and standard deviation were reported for age (years), NLR, PLR, and ESR among cases and control samples. An independent sample t-test was used to compare these mean levels between two study groups. Further, using the information on BASDAI scores for cases, data were categorized into active and inactive disease groups, and mean levels of BASDAI, ESR, NLR, and PLR were compared using independent sample t-test. P-values less than 0.05 were considered statistically significant. The graphical presentation of data was done on bar charts

## Results

Table 1 shows the mean and standard deviation of the studied parameters in both groups. The mean age of control and test group participants was the same (32 ± 4 years). In the control group, the mean NLR was 1.30 ± 0.16, the mean PLR was 94.98 ± 17.96, and the mean ESR was 16.88 ± 3.76 mm/hour. In the test group, the mean NLR was 3.08 ± 0.91, the mean PLR was 171.50 ± 38.06, and the mean ESR was 29.30 ± 9.20 mm/hour. The independent sample t-test showed there was a significant increase in mean NLR and PLR compared to the control group (p<0.05).

**Table 1:** Mean comparison of age, NLR, PLR, and ESR in cases and controls *p<0.05 was considered significant Abbreviations: NLR, neutrophil-to-lymphocyte ratio; PLR, platelet to lymphocyte ratio; ESR, erythrocyte sedimentation rate; SD, standard deviation.

Characteristics	Controls (n=59)		Cases (n=59)		p-value
	Mean	SD	Mean	SD	
Age (years)	32	4	32	4	0.95
NLR	1.30	0.16	3.08	0.91	<0.01*
PLR	94.98	17.96	171.50	39.06	<0.01*
ESR (mm/hour)	16.88	3.76	29.30	9.20	<0.01*

Table 2 compares the mean BASDAI, ESR, NLR, and PLR within cases after classification of samples on the basis of BASDAI scores into active and inactive groups. The mean BASDAI score of active disease samples was 5.91 ± 1.28, the ESR was 27.65 ± 9.07 mm/hour, NLR was 3.46 ± 0.80, and PLR was 184.39 ± 36.13. In inactive disease samples, the mean BASDAI score was 2.84 ± 0.46, the ESR was 33.42 ± 8.48 mm/hour, the NLR was 2.17 ± 0.37, and the PLR was 139.71 ± 26.05. The mean NLR and PLR were significantly higher in the active disease group (i.e., BASDAI score > 4; p<0.05). ESR showed no association with disease activity.

**Table 2:** Mean comparison of studied parameters within disease group on the basis of BASDAI *p<0.05 was considered significant Abbreviations: BASDAI, Bath Ankylosing Spondylitis Disease Activity Index; NLR, neutrophil-to-lymphocyte ratio; PLR, platelet to lymphocyte ratio; ESR, erythrocyte sedimentation rate; SD, standard deviation.

Characteristics	BASDAI ≥ 4 Active (n=42)		BASDAI < 4 Inactive (n=17)		p-value
	Mean	SD	Mean	SD	
BASDAI	5.91	1.28	2.85	0.46	<0.01*
ESR (mm/hour)	27.65	9.07	33.42	8.48	0.02*
NLR	3.46	0.80	2.17	0.37	<0.01*
PLR	184.39	36.13	139.71	26.05	<0.01*

**Figure 1:**
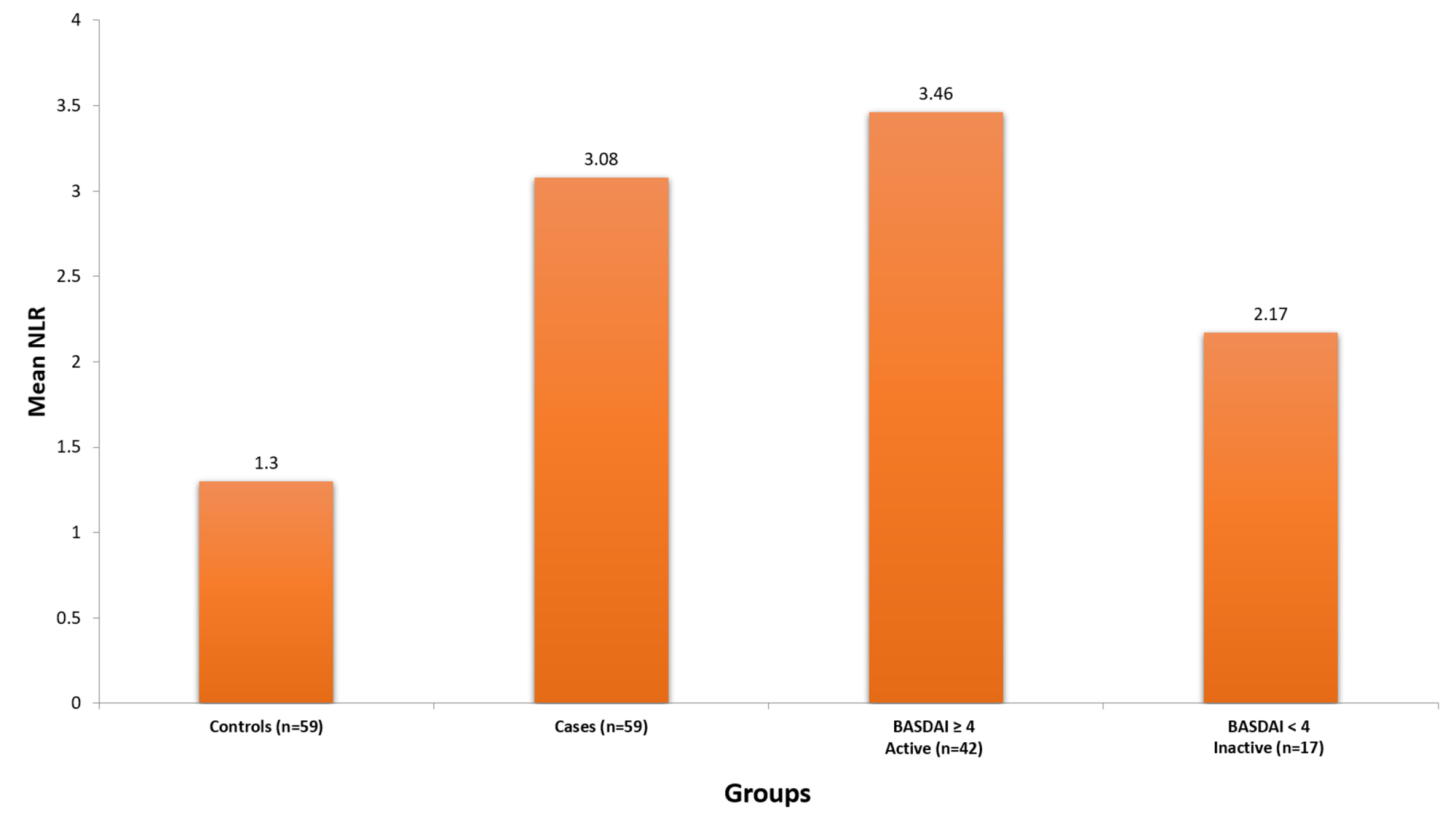
Mean neutrophil-to-lymphocyte ratio Abbreviations: BASDAI, Bath Ankylosing Spondylitis Disease Activity Index; NLR, neutrophil-to-lymphocyte ratio.

**Figure 2:**
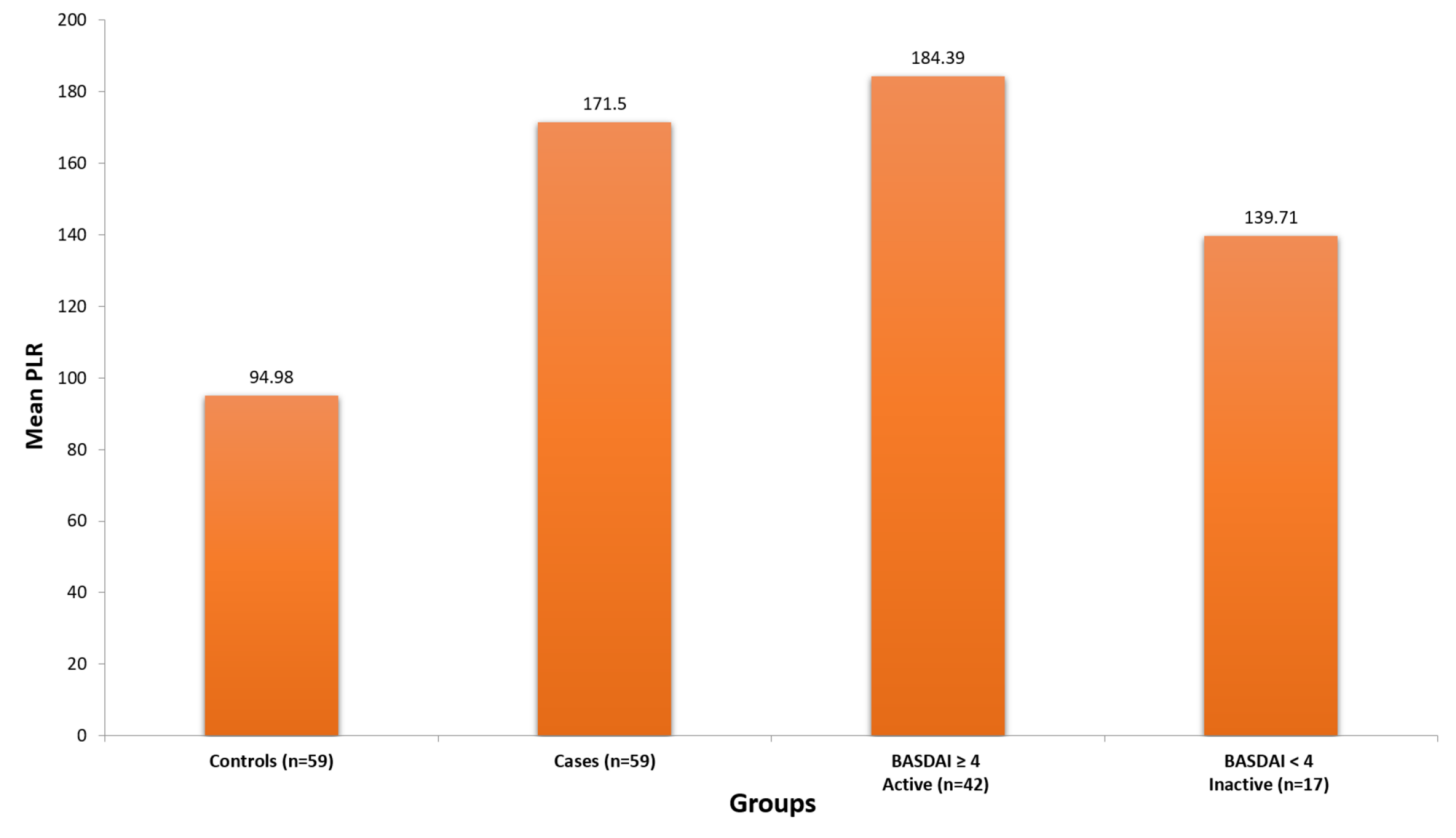
Mean platelet-to-lymphocyte ratio Abbreviations: BASDAI, Bath Ankylosing Spondylitis Disease Activity Index; PLR, platelet-to-lymphocyte ratio.

Figure 1 shows overall mean NLR, and Figure 2 shows overall mean PLR.

## Discussion

ESR is not a reliable marker for disease activity in AS [10]. In a study by Spoorenberg et al., ESR and C-reactive protein (CRP) were not good inflammatory markers for disease activity in AS with spine disease [14]. Our study showed that ESR had no relationship with AS activity status.

NLR and PLR are considered good inflammatory markers and may be used for assessing disease activity in SLE as shown by some studies (Poster: Gunawan H, Awalia A, Soeroso J. 159 Neutrophil-to-Lymphocyte Ratio and Systemic Lupus Erythematosus: A new Parameter for Disease Activity Assessment?; 2017) [15]. NLR can be used for disease activity and differentiation of infection in SLE (Poster: Aponte J, Carrizosa J, Sanchez A, Ospina M, Cartagena A, Zapata C, Cervera R. 202 The Role of Neutrophil-Lymphocyte Ratio (NLR), and Other Biomarkers (C-Reactive Protein CRP, Count of Monocytes and Lymphocytes) Differentiating Lupic Activity (FLARE) from Infection; 2017). Some studies showed that NLR and PLR are associated with active RA patients, and NLR helps in the early diagnosis of RA [16,17]. A study by Mercan et al. showed high NLR in RA and AS patients was associated with active RA [18]. High NLR and PLR was noted in AS patients compared with control. We also found higher NLR and PLR in AS patients with active AS (i.e., BASDAI score > 4).

NLR and PLR can be easily calculated by dividing the neutrophil and platelets counts by the number of lymphocytes, respectively. FBC is a simple, cost-effective routine test. As suggested by multiple studies, NLR and PLR may be considered as markers of disease status in RA, SLE, Takayasu arteritis, ulcerative colitis, other autoimmune diseases, and infection [19-23]. NLR and PLR are emerging markers of inflammatory conditions and disease activity. NLR and PLR, as indices, have reduced risk for observer error as compared to clinical activity scores like BASDAI for AS. Higher NLR and PLR were associated with clinically active AS. NLR and PLR may be used for monitoring AS in developing countries.

Our study was limited in that it was a single-center study and represents a cohort of patients belonging to the same ethnicity. A multi-center study with a wider sampling of patients would address these limitations.

## Conclusions

We conducted this case-control study to determine the association of the NLR and PLR with the disease activity of AS, a chronic rheumatological condition affecting the sacroiliac joint and spine of young patients. According to our findings, mean NLR and PLR were higher in AS patients compared to healthy controls, and high NLR and PLR values also correlated to disease activity levels. Therefore, NLR and PLR are good markers of inflammation and disease activity in AS patients. Given that NLR and PLR can be easily calculated from FBC, an inexpensive, easily available test, NLR and PLR are promising markers to assess disease activity and help treatment optimization in AS patients.
